# Supplementary figures and images for: Single position substitution of hairpin pyrrole-imidazole polyamides imparts distinct DNA-binding profiles across the human genome
Source: PLoS One. 2020 Dec 22;15(12):e0243905. doi: 10.1371/journal.pone.0243905 (PMC7755219; doi:10.1371/journal.pone.0243905)

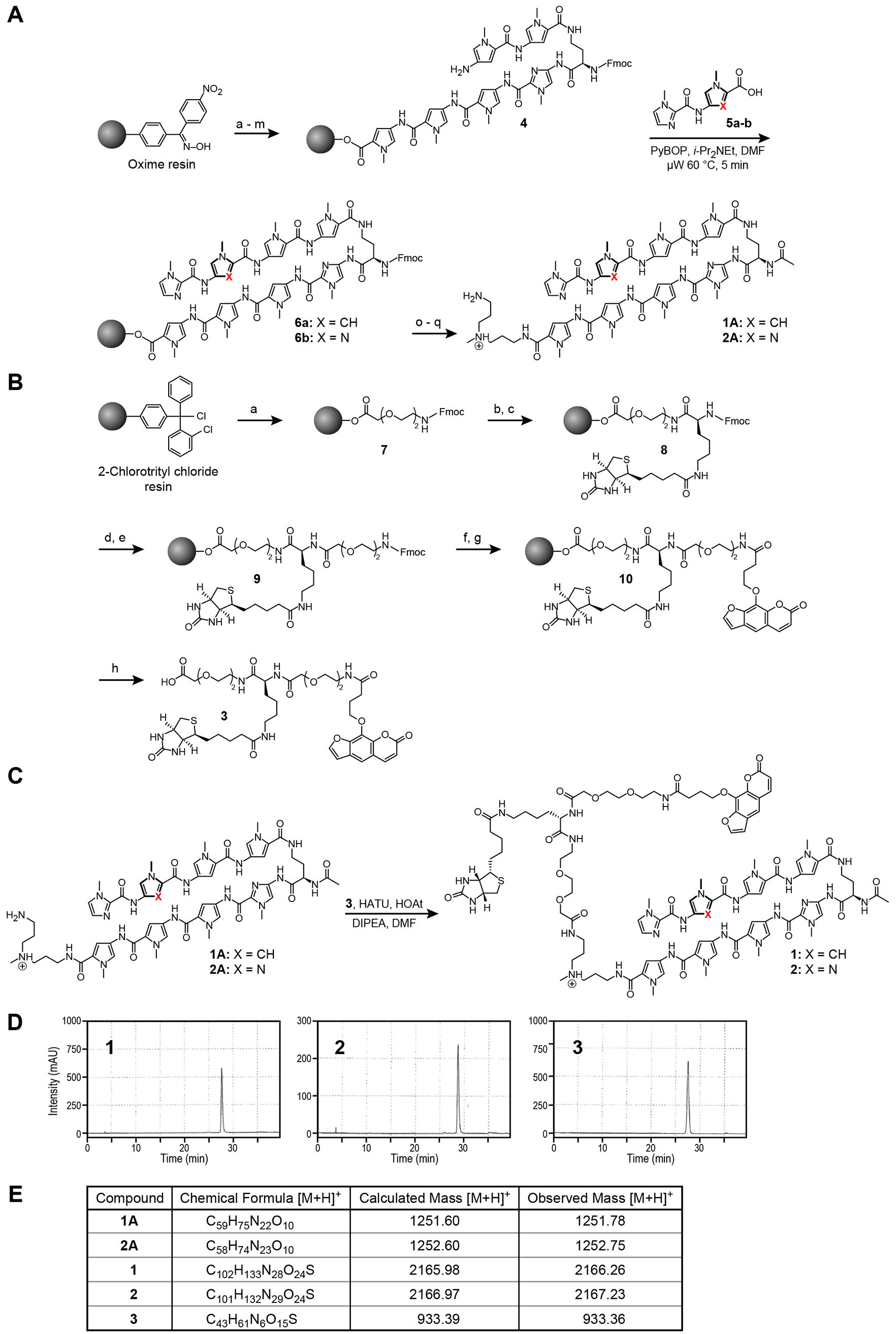

Supplement: S1 Fig — (A) Solid phase synthetic scheme for the synthesis of Py-Im polyamides 1A and 2A, a) Boc-Py-OBt, i-Pr2NEt, DMF, μW (80°C, 3 h); b) 80:1:19 TFA:triethylsilane:CH2Cl2, 5 min, RT; c) Boc-Py-OH, PyBOP i-Pr2NEt, DMF, μW (60°C, 5 min); d) 9:2:1 DMF:Ac2O:i-Pr2NEt, 30 min, RT; e) repeat (1x) steps b–d; f) 80:1:19 TFA:triethylsilane:CH2Cl2, 5 min, RT; g) Boc-Im-OH, PyBOP, i-Pr2Net, DMF, μW (60°C, 5 min); h) 9:2:1 DMF:Ac2O:i-Pr2NEt, 30 min, RT; i) 80:1:19 TFA:triethylsilane:CH2Cl2, 25 min, RT; j) Fmoc-D-Dab(Boc)-OH, PyBOP, i-Pr2Net, DMF, μW (60°C, 25 min); k) 9:2:1 DMF:Ac2O:i-Pr2NEt, 30 min, RT; l) repeat (2x) steps b–d; m) 80:1:19 TFA:triethylsilane:CH2Cl2, 5 min, RT; n) 5, PyBOP, i-Pr2NEt, DMF, μW (60°C, 5 min); o) 20% piperidine, DMF, 30 min, RT; p) 9:2:1, DMF:Ac2O:i-Pr2NEt 30 min, RT; q) neat 3,3′-Diamino-N-methyldipropylamine, μW (60°C, 10 min); (B) Synthesis of the psoralen–biotin-acid moiety 3, a) Fmoc-PEG2-OH, i-Pr2NEt, DCM; b) 20% piperidine, DMF; c) Biotin-Lys(Fmoc)-OH, HATU, HOAt, i-Pr2NEt, 3:1 DMSO:DMF; d) 20% piperidine, DMF; e) Fmoc-PEG2-OH, HATU, HOAt, i-Pr2NEt, DMF; f) 20% piperidine, DMF; g) SPB (NHS-psoralen), i-Pr2NEt, DMF; h) 95% TFA, 2.5% H2O, 2.5% i-Pr3SiH; and (C) peptide coupling of Py-Im polyamides 1A and 2A with 3; (C) Analytical HPLC traces of 1, 2, and 3; (D) Characterization of compounds by MALDI-TOF. (TIF) [file pone.0243905.s001.tif]

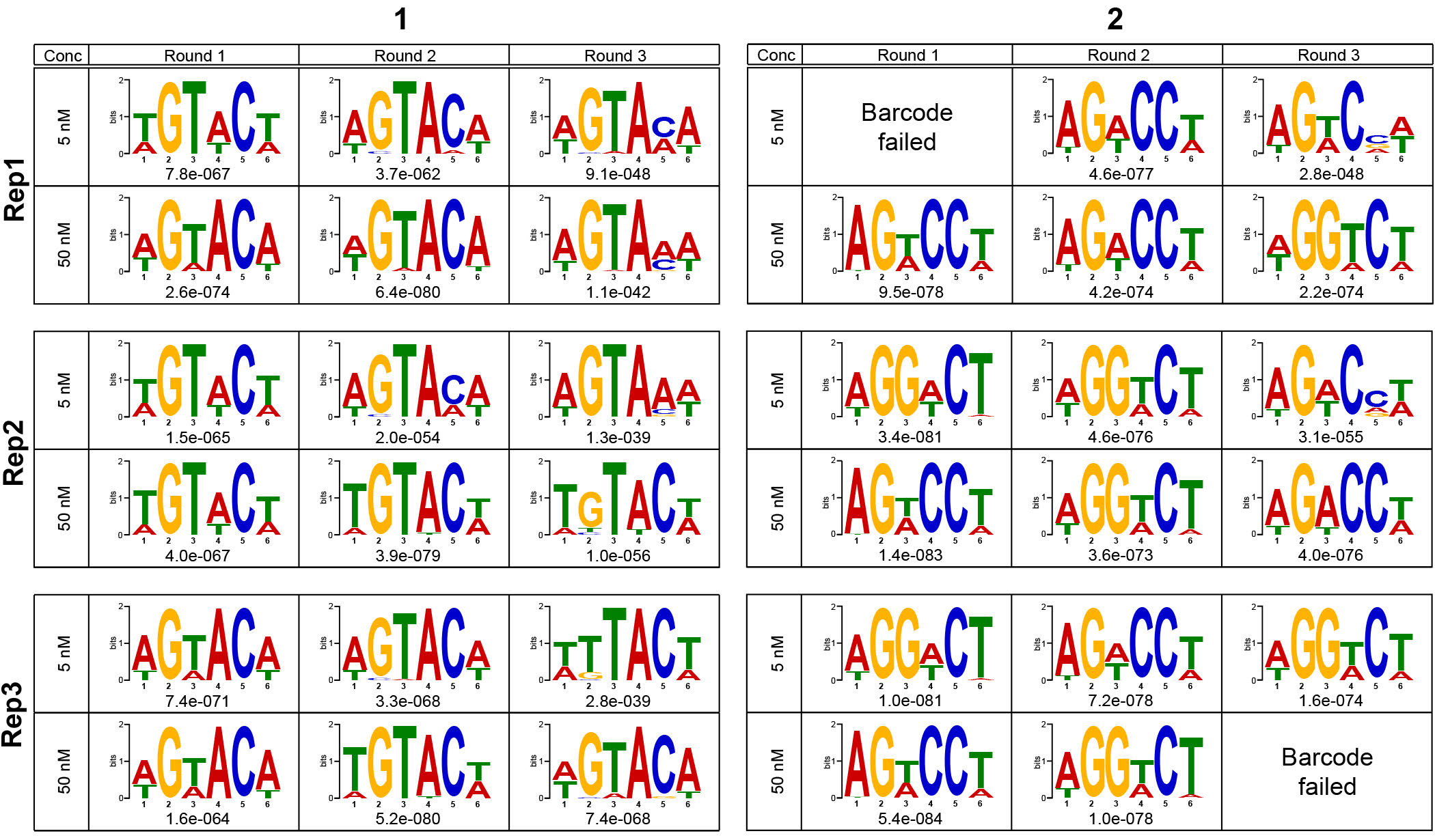

Supplement: S2 Fig — PWMs for three replicates of 1 (left) and 2 (right) at two concentrations (5 nM and 50 nM) and three enrichment rounds (1, 2 and 3). The PWMs are derived using MEME software with the corresponding e-value indicated. (TIF) [file pone.0243905.s002.tif]

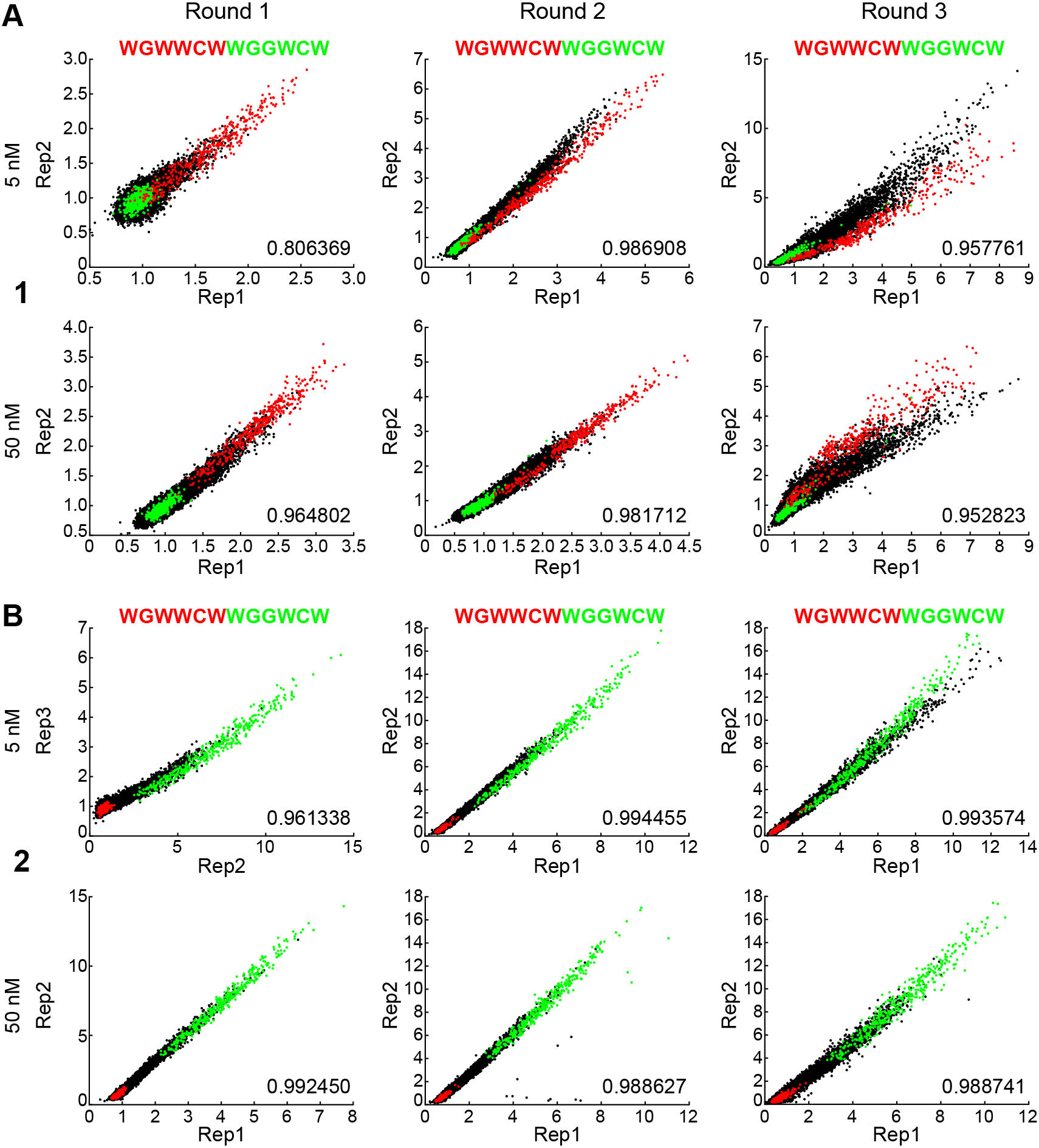

Supplement: S3 Fig — The height of each peak corresponds to CSI enrichment for a given sequence. (A) SEL for 1 with seed motif WGWWCW (where W = A or T). (B) Top view of the SEL in A. (C) SEL for 2 with seed motif WGGWCW (where W = A or T). (D) Top view of the SEL in C. (E) SELs consists of concentric rings with sequences in the 0 mismatch ring (central ring) having an exact match to the seed motif. Moving outwards, the 1 mismatch ring contains all sequences that differ from the seed motif at any one position (or a Hamming distance of one). In each ring, sequences are arranged clockwise by position of the mismatch, then alphabetically by the sequence. The 1 mismatch ring begins with mismatches at the first position of the motif and ends with mismatches at the last position of the motif. (TIF) [file pone.0243905.s003.tif]

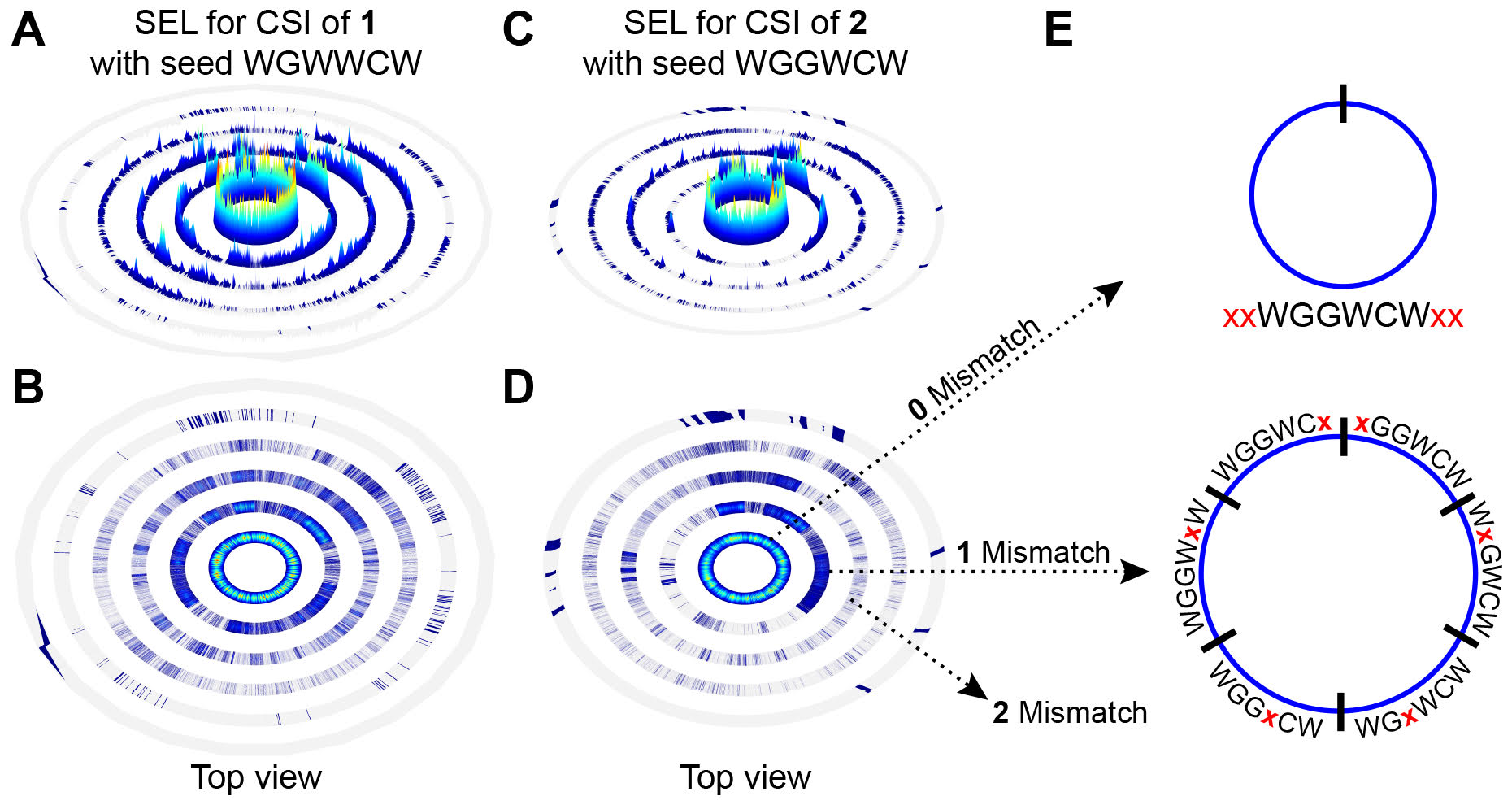

Supplement: S4 Fig — SELs for three replicates of 1 (left) and 2 (right) at two concentrations (5 nM and 50 nM) and three enrichment rounds (1, 2 and 3). (TIF) [file pone.0243905.s004.tif]

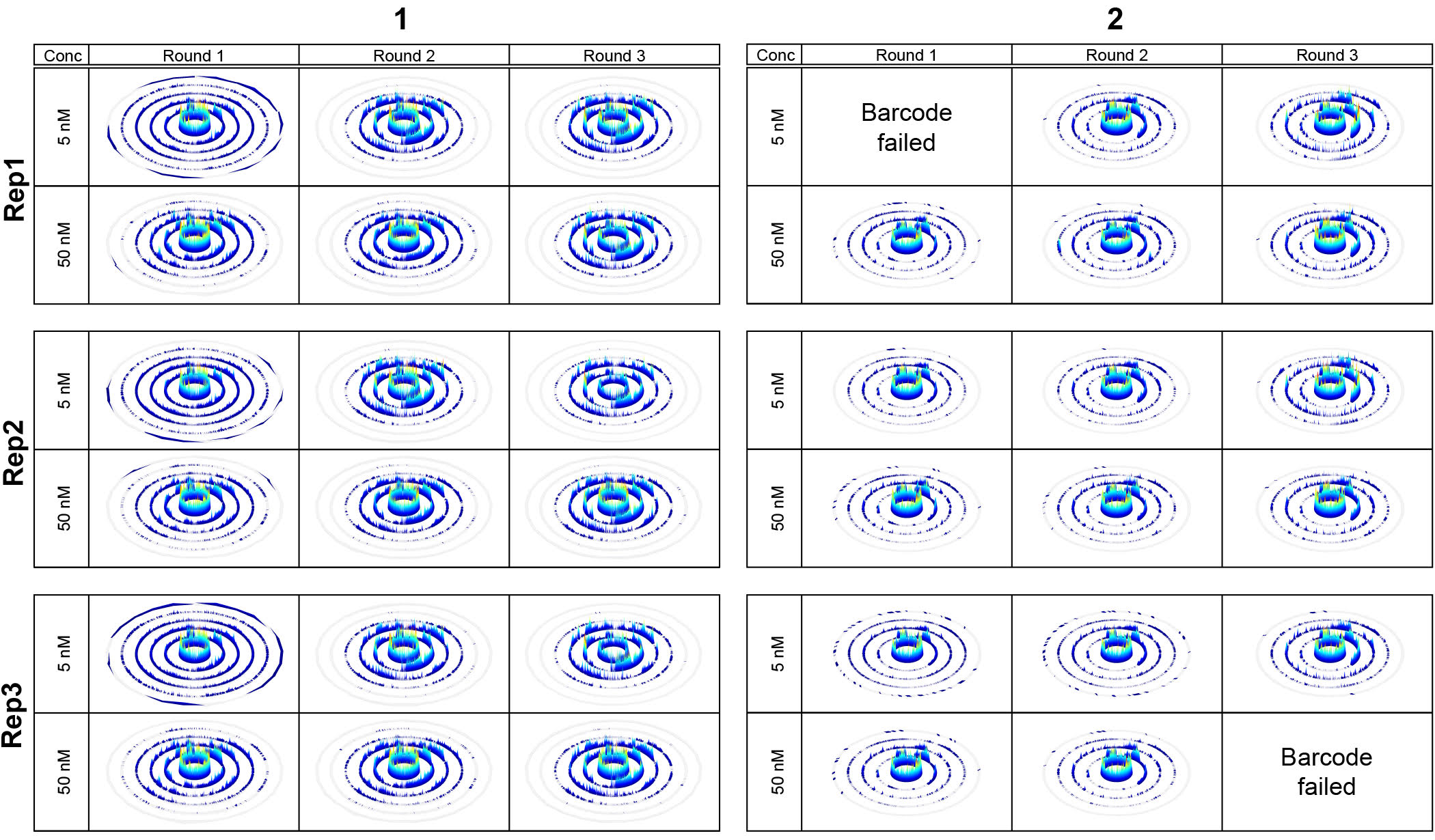

Supplement: S5 Fig — Scatter plots for CSI enrichment of 1 (A) and 2 (B) at two concentrations (5 nM and 50 nM) and three enrichment rounds (1, 2 and 3). (TIF) [file pone.0243905.s005.tif]

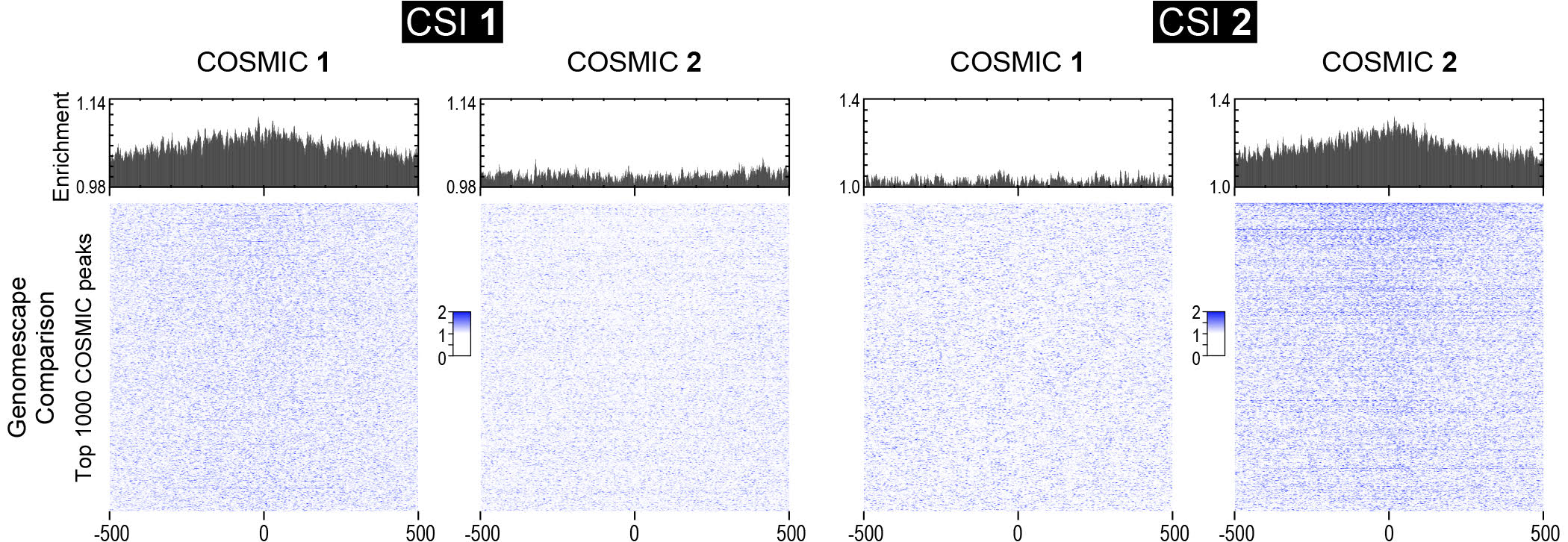

Supplement: S6 Fig — Data is displayed as averaged bar plots (top) and heatmap of genomescapes (bottom) for top 1000 COSMIC peaks mapped on a 1 Kbp region. CSI data from enrichment round 1 at 50 nM for 1 (left) and 2 (right) was used for genomescape generation. (TIF) [file pone.0243905.s006.tif]

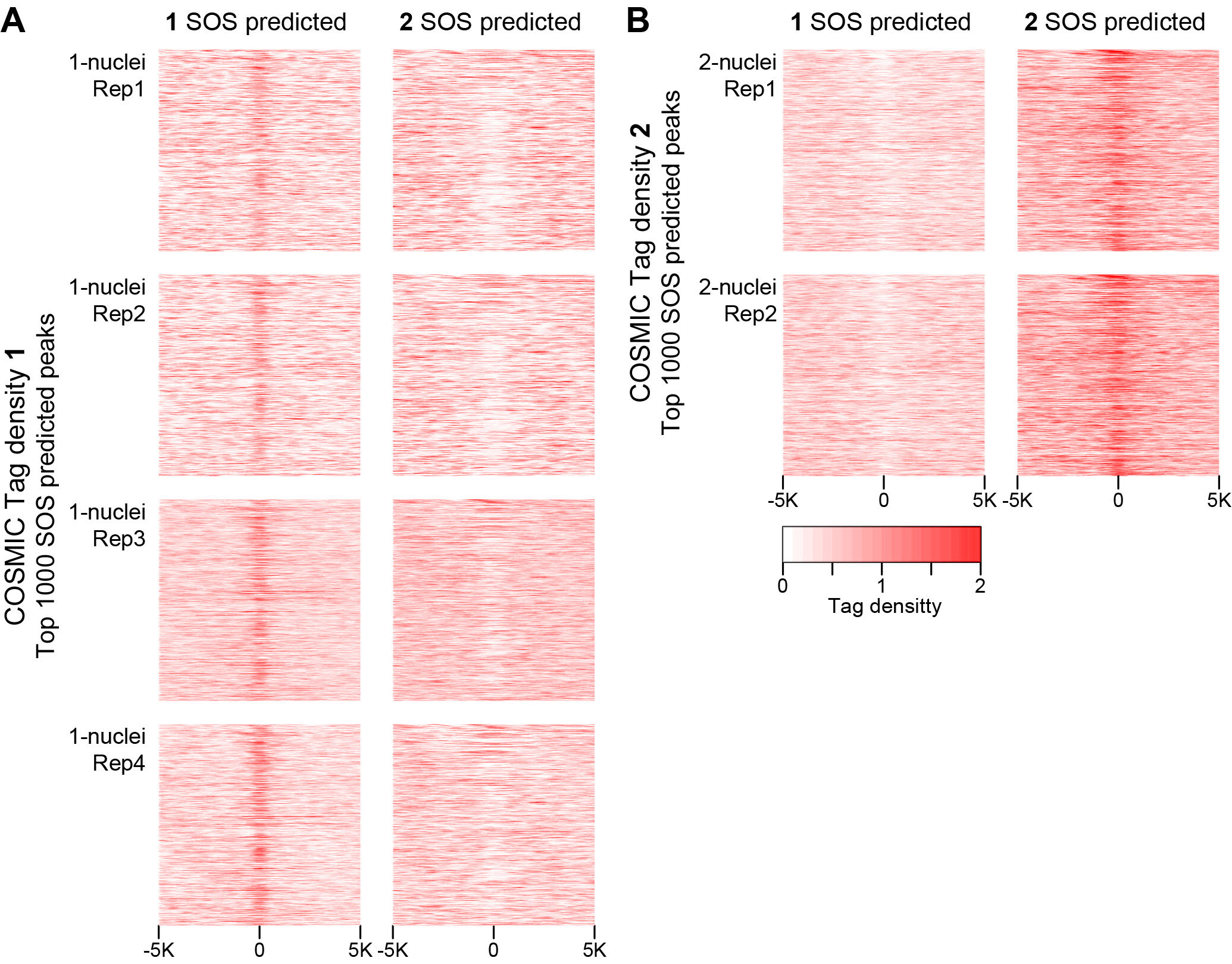

Supplement: S7 Fig — Heatmaps with tag density for COSMIC replicates of 1 (A) and 2 (B) are mapped for the top 1000 SOS predicted genomic peaks using a 10 Kbp window. CSI data from enrichment round 1 at 50 nM for 1 (left) and 2 (right) was used for SOS prediction. (TIF) [file pone.0243905.s007.tif]

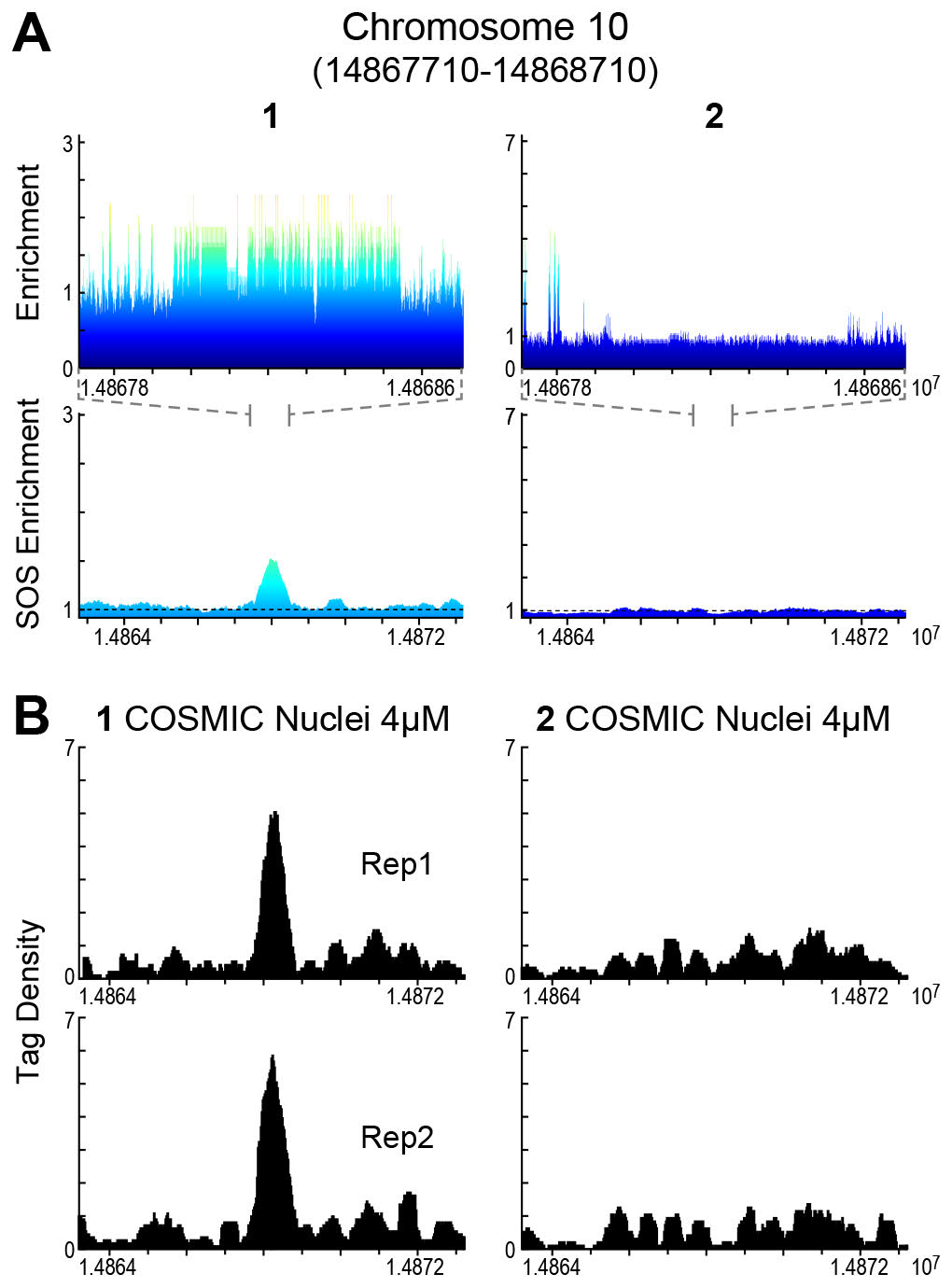

Supplement: S8 Fig — (A) Genomescapes (top) displaying a 1 Kbp region and SOS enrichment plots (bottom) displaying a 10 Kbp region for polyamides 1 and 2 at genomic loci of chr10. (B) COSMIC tag density data of replicates of 1 and 2 for a 10 Kbp region at same loci. (TIF) [file pone.0243905.s008.tif]

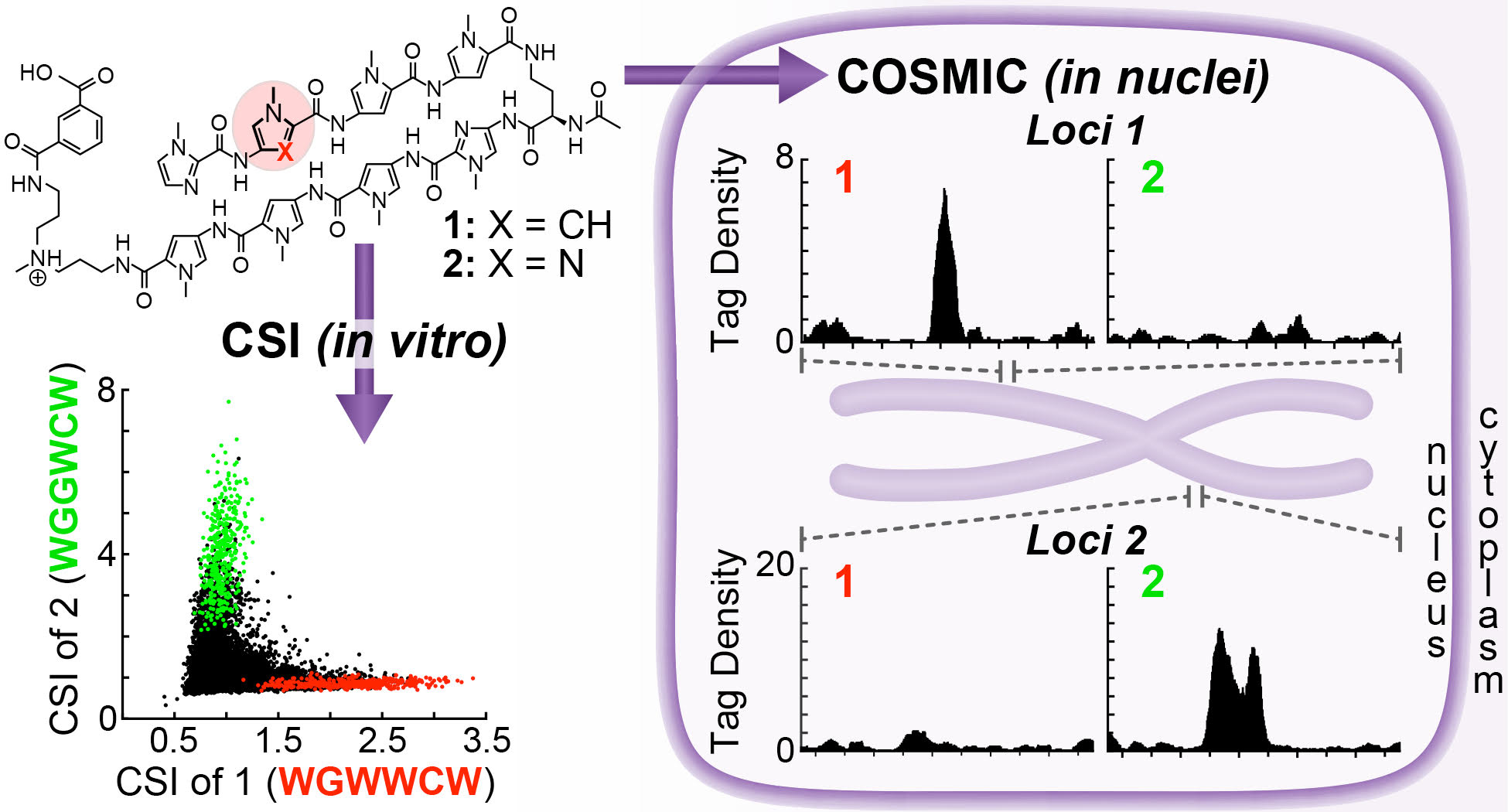

Supplement: S9 Fig — (TIFF) [file pone.0243905.s009.tiff]
