# Supplementary material for: Single position substitution of hairpin pyrrole-imidazole polyamides imparts distinct DNA-binding profiles across the human genome
Source: PLoS One. 2020 Dec 22;15(12):e0243905. doi: 10.1371/journal.pone.0243905 (PMC7755219; doi:10.1371/journal.pone.0243905)
Supplement: S1 File — Heatmaps for SOS and genomescape data for enrichment round 1 at 50 nM for polyamide 1 and 2. Heatmaps are plotted for the top 1000 COSMIC peaks of COSMIC replicates of polyamides 1 and 2 on a 10 Kbp window for SOS and 1 Kbp for genomescapes. (PDF) [file pone.0243905.s010.pdf]

SOS and GENOMESCAPE Heatmap at top 1000 COSMIC sites

Contents

1 1-nuclei-Rep1 **2**

2 1-nuclei-Rep2 **3**

3 1-nuclei-Rep3 **4**

4 1-nuclei-Rep4 **5**

5 2-nuclei-Rep1 **6**

6 2-nuclei-Rep2 **7**

1 1-nuclei-Rep1

CSI FILE	SOS heatmap	Genomescape
1-RND1-50nM	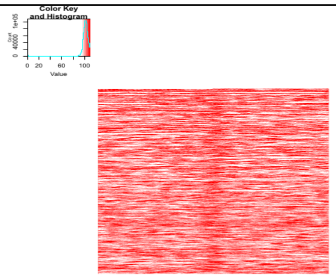	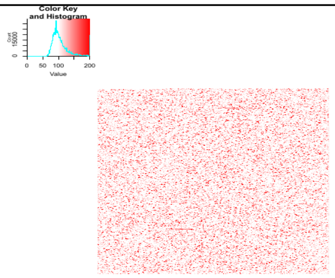
2-RND1-50nM	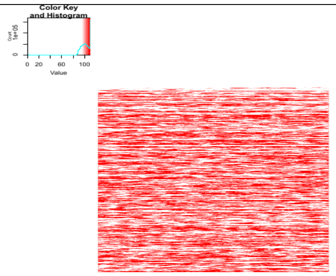	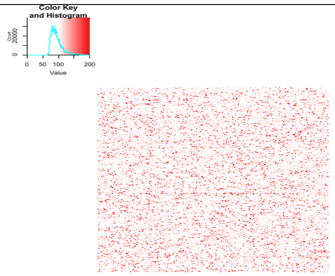

2 1-nuclei-Rep2

CSI FILE	SOS heatmap	Genomescape
1-RND1-50nM	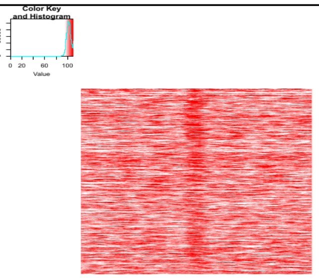	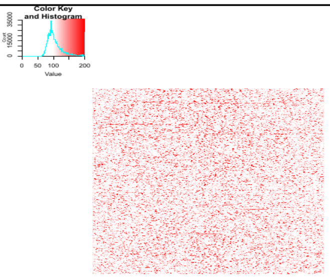
2-RND1-50nM	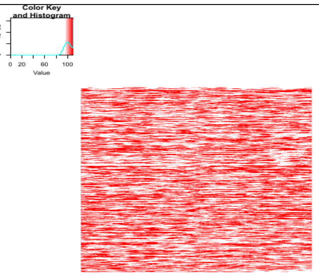	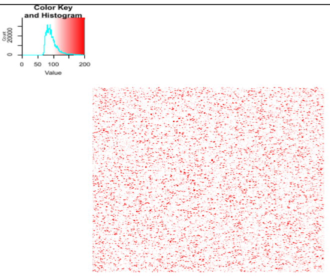

3 1-nuclei-Rep3

CSI FILE	SOS heatmap	Genomescape
1-RND1-50nM	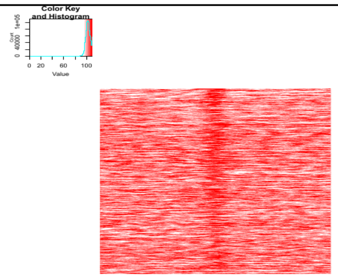	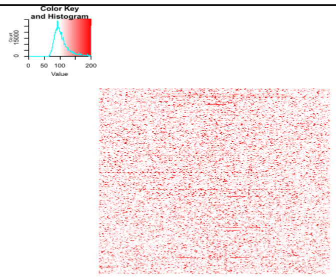
2-RND1-50nM	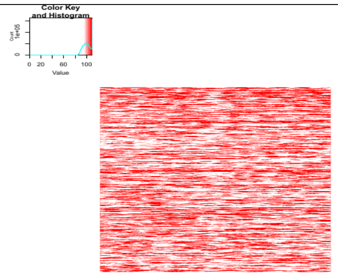	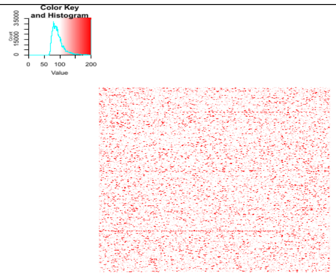

4 1-nuclei-Rep4

CSI FILE	SOS heatmap	Genomescape
1-RND1-50nM	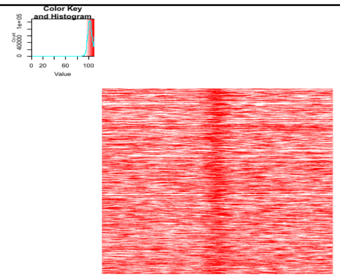	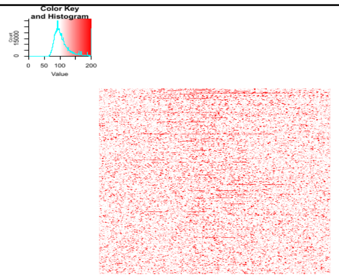
2-RND1-50nM	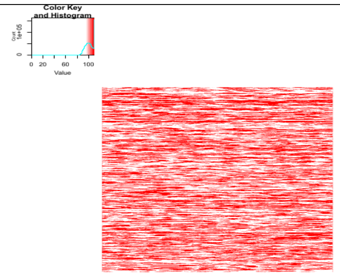	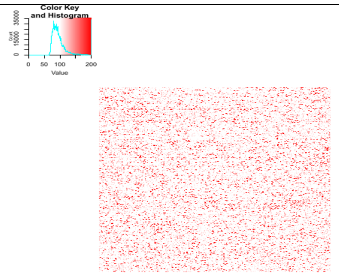

5 2-nuclei-Rep1

CSI FILE	SOS heatmap	Genomescape
1-RND1-50nM	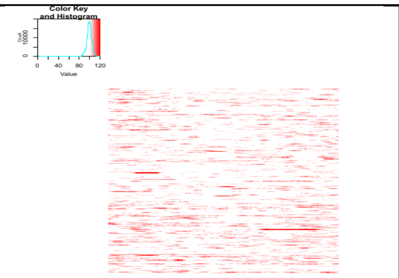	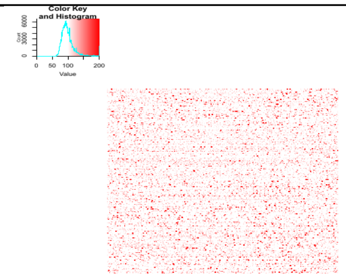
2-RND1-50nM	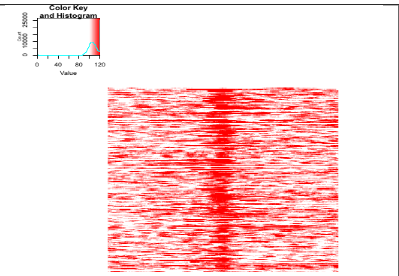	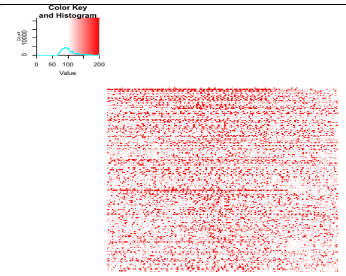

6 2-nuclei-Rep2

CSI FILE	SOS heatmap	Genomescape
1-RND1-50nM	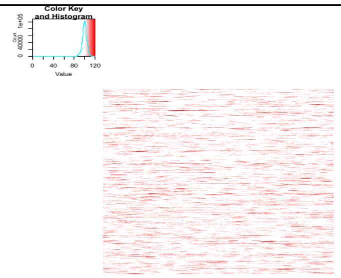	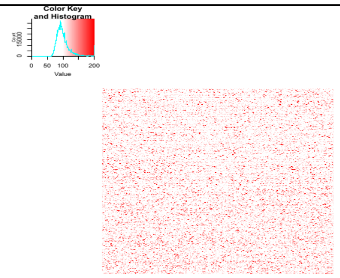
2-RND1-50nM	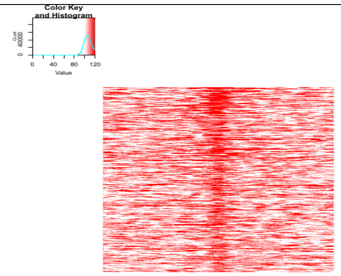	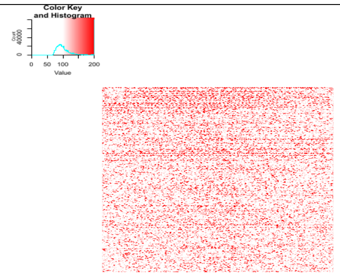
