# Supplementary material for: Single position substitution of hairpin pyrrole-imidazole polyamides imparts distinct DNA-binding profiles across the human genome
Source: PLoS One. 2020 Dec 22;15(12):e0243905. doi: 10.1371/journal.pone.0243905 (PMC7755219; doi:10.1371/journal.pone.0243905)
Supplement: S2 File — Tag density heatmaps for polyamide 1 and 2 replicates are mapped for the top 1000 SOS predicted genomic peaks using a 10 Kbp window. CSI data from enrichment round 1 at 50 nM for 1 and 2 was used for SOS prediction. (PDF) [file pone.0243905.s011.pdf]

COSMIC tag density Heatmap at top 1000 SOS predicted sites

Contents

1 **WGWWCW-RND1-C50nM** **2**

2 **WGGWCW-RND1-C50nM** **3**

1 WGWWCW-RND1-C50nM

COSMIC FILE	Tag density Heatmap
1-nuclei-Rep1	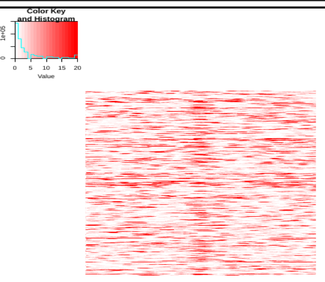
1-nuclei-Rep2	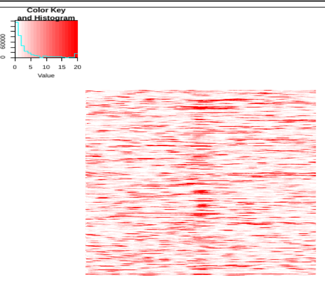
1-nuclei-Rep3	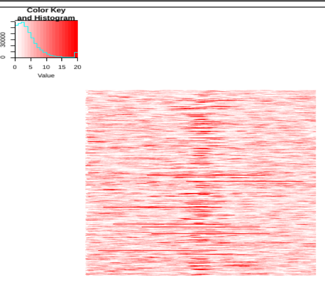
1-nuclei-Rep4	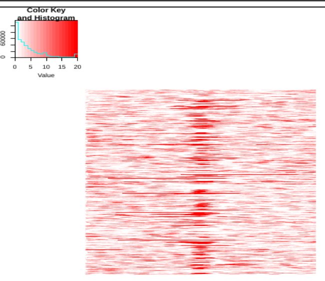
2-nuclei-Rep1	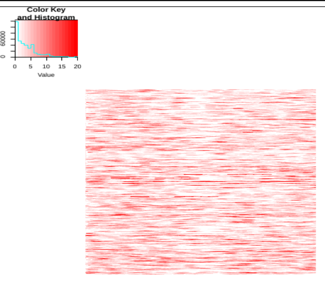
2-nuclei-Rep2	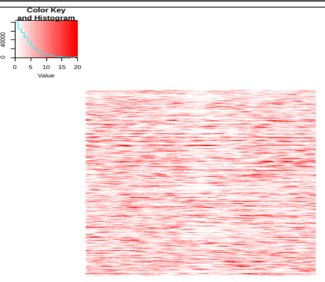

2 WGGWCW-RND1-C50nM

COSMIC FILE	Tag density Heatmap
1-nuclei-Rep1	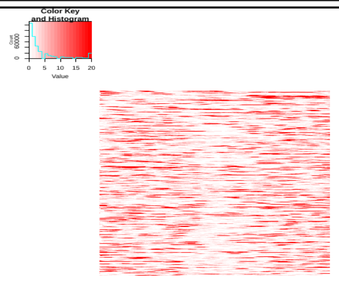
1-nuclei-Rep2	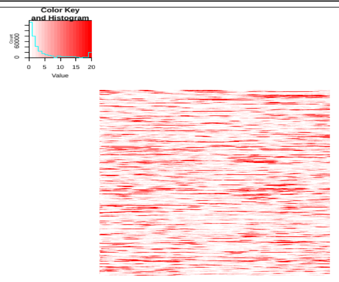
1-nuclei-Rep3	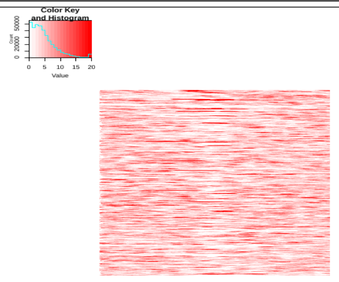
1-nuclei-Rep4	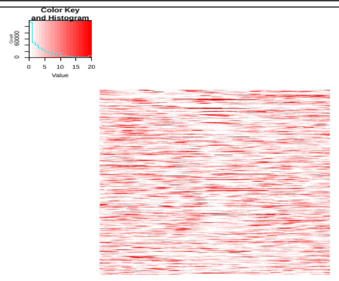
2-nuclei-Rep1	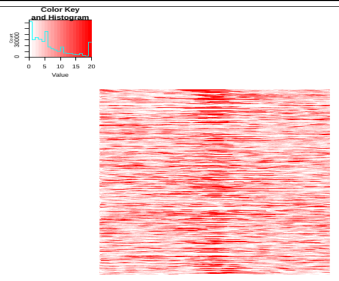
2-nuclei-Rep2	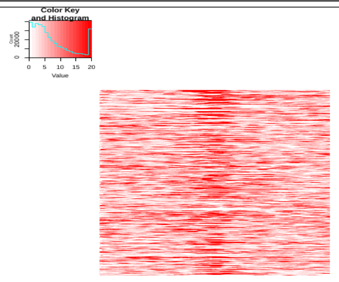
